# New Emerging Chemokine Receptors: CCR5 or CXCR5 on Tumor Is Associated with Poor Response to Chemotherapy and Poor Prognosis in Locally Advanced Triple-Negative Breast Cancer

**DOI:** 10.3390/cancers16132388

**Published:** 2024-06-28

**Authors:** Neslihan Cabioglu, Semen Onder, Hüseyin Karatay, Aysel Bayram, Gizem Oner, Mustafa Tukenmez, Mahmut Muslumanoglu, Abdullah Igci, Ahmet Dinccag, Vahit Ozmen, Adnan Aydiner, Pınar Saip, Ekrem Yavuz

**Affiliations:** 1 Department of General Surgery, Istanbul Faculty of Medicine, Istanbul University, Istanbul 34452, Turkey; onergizem@gmail.com (G.O.); mustafatukenmez@hotmail.com (M.T.); mahmutm@istanbul.edu.tr (M.M.); aigci@istanbul.edu.tr (A.I.); ahmet.dinccag@istanbul.edu.tr (A.D.); vozmen@istanbul.edu.tr (V.O.); 2 Department of Pathology, Istanbul Faculty of Medicine, Istanbul University, Istanbul 34452, Turkey; semen_yesil@yahoo.com.tr (S.O.); huseyin140@gmail.com (H.K.); aysel.bayram@istanbul.edu.tr (A.B.); ekremyavuz2006@gmail.com (E.Y.); 3 Department of Medical Oncology, Institute of Oncology, Istanbul University, Istanbul 34452, Turkey; adnanaydiner@superonline.com (A.A.); pinarsaip@gmail.com (P.S.)

**Keywords:** triple-negative breast cancer, CCR5, CCR7, CXCR4, CXCR5, tumor-infiltrating lymphocytes, neoadjuvant chemotherapy

## Abstract

**Simple Summary:**

We investigated any possible associations between chemokine receptor expression and responses to chemotherapy along with survival outcomes in patients with triple-negative breast cancer with locally advanced disease. Expressions of chemokine receptors were examined after staining surgical specimens using specific antibodies for CCR5, CCR7, CXCR4, and CXCR5. Patients with high CCR5, CCR7, CXCR4, and CXCR5 expression on tumors and high CXCR4 expression on tumor-infiltrating lymphocytes were less likely to have a chemotherapy response compared to others. Patients with residual lymph node metastases after chemotherapy, CCR5, or CXCR4 expressions on tumors have shown a worse outcome compared to others. However, patients with CXCR5 expression on tumor-infiltrating lymphocytes had a better outcome compared to those without. High expression of CXCR4 or CCR5 on tumor was found to be associated with poor prognosis, and CXCR5 on tumor was associated with poor chemotherapy response in the present cohort with locally advanced triple-negative breast cancer. Our results suggest that patients with triple-negative breast cancer could benefit from a chemokine receptor inhibitor therapy.

**Abstract:**

Background: We aim to investigate any possible associations between chemokine receptor expression and responses to neoadjuvant chemotherapy (NAC) along with outcomes in patients with triple-negative breast cancer (TNBC) with locally advanced disease. Method: Expressions of chemokine receptors were examined immunohistochemically after staining archival tissue of surgical specimens (n = 63) using specific antibodies for CCR5, CCR7, CXCR4, and CXCR5. Results: Patients with high CCR5, CCR7, CXCR4, and CXCR5 expression on tumors and high CXCR4 expression on tumor-infiltrating lymphocytes (TILs) were less likely to have a pathological complete response (pCR) or Class 0-I RCB-Index compared to others. Patients with residual lymph node metastases (ypN-positive), high CCR5_TM(tumor)_, and high CXCR4_TM_ expressions had an increased hazard ratio (HR) compared to others (DFS: HR = 2.655 [1.029–6.852]; DSS: HR = 2.763 [1.008–7.574]), (DFS: HR = 2.036 [0.805–5.148]; DSS: HR = 2.689 [1.020–7.090]), and (DFS: HR = 2.908 [1.080–7.829]; DSS: HR = 2.132 (0.778–5.846)), respectively. However, patients without CXCR5_TIL_ expression had an increased HR compared to those with CXCR5_TIL_ (DFS: 2.838 [1.266–6.362]; DSS: 4.211 [1.770–10.016]). Conclusions: High expression of CXCR4_TM_ and CCR5_TM_ was found to be associated with poor prognosis, and CXCR5_TM_ was associated with poor chemotherapy response in the present cohort with locally advanced TNBC. Our results suggest that patients with TNBC could benefit from a chemokine receptor inhibitor therapy containing neoadjuvant chemotherapy protocols.

## 1. Background

Triple-negative breast cancer (TNBC) constitutes about 15–20% of all diagnosed breast cancers [1,2,3]. Although TNBC is typically characterized by a high degree of aggressiveness and poor prognosis, no approved targeted molecular therapies for this type of cancer have yet been developed [4,5]. Novel targeted molecular therapies are under investigation for patients with TNBC who are resistant to neoadjuvant chemotherapy (NAC) or those with a partial response to NAC.

Furthermore, TNBC has been proven to be the most immunogenic tumor type among other breast cancer subtypes that were described by immunochemistry (IHC) staining according to the expression of estrogen or progesterone receptors or HER2 [6]. The presence of an immune-rich tumor microenvironment, as described by the presence of tumor-infiltrating lymphocytes, was found to be associated with improved survival and with a benefit from immunotherapeutic approaches, including immune checkpoint inhibitor therapies [2,7].

The CXCR4 receptor is the most studied of all chemokine receptors in cancer. Its roles in breast cancer include inducing cell survival, proliferation, motility, invasion, angiogenesis, recruitment, and metastasis [8,9,10]. The chemokine receptors family could be the biological support of the ‘seed and soil’ theory [10]. CXCR4 was more likely to be expressed in bone and bone marrow metastases than visceral metastases [11,12]. CCR7 is often up-regulated together with CXCR4 in cancer and is associated with lymph node metastases in breast cancer [8,13,14]. In node-positive breast cancer, expression of chemokine receptors was found to be associated with an increased risk of relapse in certain organs. Expressions of CXCR4 and CCR7 were associated with an increased risk of metastasis to the liver and skin, respectively, whereas expressions of CX3CR1 and CCR6 were associated with metastasis to the brain and pleura [15]. Previous studies have demonstrated high levels of chemokine receptor expression in inflammatory breast cancer (IBC) and early breast cancer associated with metastatic spread [16,17].

There are few articles about the role of new chemokine receptors, including CXCR5 and CCR5, in breast cancer. The high expression of CXCL13 and CXCR5 in breast cancer tissue was found to be associated with lymph node metastasis, distant metastasis, and a more advanced disease stage [18]. Similarly, expression of CCR5 was associated with increased tumor growth that could be inhibited with a CCR5 inhibitor maraviroc [19].

Different mechanisms were described regarding the CXCR4–CXCL12 axis to induce metastasis [9]. CXCL12 was shown to transactivate HER2-neu in the breast cancer cell lines MDA-MB-361 and SKBR3, which express both CXCR4 and HER2-neu that was inhibited by AMD3100, a CXCR4 inhibitor, PKI 166, an epidermal growth factor receptor/HER2- tyrosine kinase inhibitor, and PP2, a Src kinase inhibitor. Furthermore, targeting CXCR4 with AMD3100 suppressed tumor growth in vitro and in vivo and synergized with docetaxel in trastuzumab-resistant cell lines and in a trastuzumab-resistant xenograft mouse model [20].

However, there is little data about chemokine receptor expression and its role in cancer progression and outcome of patients with TNBC. These previous studies have indicated some associations between CXCR4, CCR7, and CCR5 expression in TNBC and poor prognosis [8,21,22,23]. The knockdown of CCR7 decreased tumor cell proliferation, migration, and invasion in vitro using the TNBC cell lines, 4T1 and MDA-MB-231, and reduced the distant metastasis of 4T1 cells in an orthotopic mouse model [22].

Programmed death ligand 1 (PD-L1) is expressed in 20% of TNBCs, suggesting PD-L1 is a therapeutic target in TNBCs [24]. Important advances have been achieved in cancer therapy with immune checkpoint inhibitors in TNBC with inhibition of PD-L1 [24,25,26]. In a recently published phase 3 trial, the addition of PD-L1 inhibitor pembrolizumab to chemotherapy resulted in longer progression-free survival than chemotherapy alone among patients with advanced triple-negative breast cancer whose tumors expressed higher levels of PD-L1 [26]. Similarly, inhibition or blockade of the chemokine–chemokine receptor cascade might provide alternative therapeutic options for future trials, especially for patients with residual TNBC following NAC [27,28,29].

In this study, we aim to investigate any association between chemokine receptor expression and responses to chemotherapy along with the survival of patients with TNBC who presented with locally advanced disease and then received NAC followed by surgery. Targeting the chemokine–chemokine receptor axis in residual cancer cells following NAC in an adjuvant setting might further improve the outcome of patients with an incomplete response to chemotherapy.

## 2. Methods

### 2.1. Patients and Tissue Material

**Figure 1:**
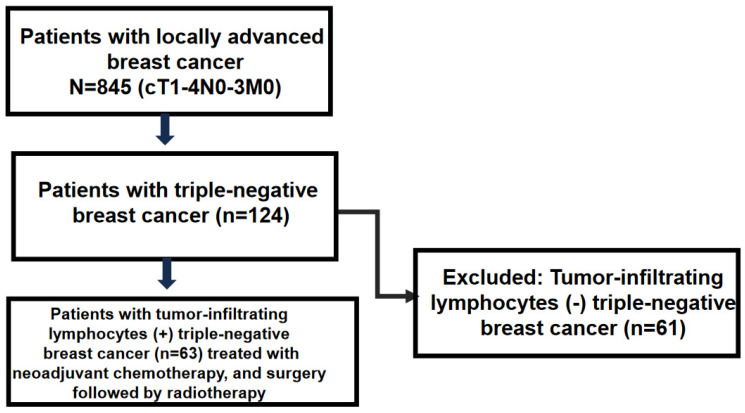
Study cohort.

Between July 2002 and December 2017, 852 patients underwent NAC at Istanbul University, Faculty of Medicine Department of General Surgery. Based on the results of the previous pathology reports, patients who underwent surgery following NAC due to locally advanced breast cancer (LABC) were included in the study. Demographic and tumor characteristics and follow-up time were analyzed retrospectively. Patients with male breast cancer, pregnancy-associated breast cancer, and bilateral breast cancers, and those with distant metastases were excluded from the analysis. Patients with a core biopsy indicating triple-negative breast cancer were determined. Of those, patients with tumor-infiltrating lymphocytes (TILs) were included in the study (Figure 1). Patient and tumor characteristics were analyzed to evaluate the clinicopathological factors and outcomes in the study group. Ethical committee approval was obtained from the Istanbul University, Istanbul Faculty of Medicine.

Estrogen and progesterone receptors and human epidermal growth factor 2 (HER2) were examined immunohistochemically (IHC). Patients with TNBC were selected based on the results of previous pathology reports. The absence of a residual invasive tumor in the resected breast specimen and in the regional lymph nodes after completion of NAC was defined as pCR. The MD Anderson Cancer Center Residual Cancer Burden Index was calculated to measure chemotherapy responses based on the size and characteristics of the residual invasive tumor and lymph node metastases using this calculator (https://www3.mdanderson.org/app/medcalc/index.cfm?pagename=jsconvert3, accessed on 24 June 2024), as described previously [30]. The calculated Residual Cancer Burden (=RCB) scores obtained after neoadjuvant treatment were further classified as 0, I, II, and III, where “0” was considered “pCR”, “I” was similarly associated with a good chemotherapy response, and “II” and “III” were associated with worse chemotherapy responses.

### 2.2. Immunohistochemical Evaluation

Immunological markers were studied in retrospectively collected archival tissue material of surgical specimens (n = 63) based on immunohistochemistry. Those tumor paraffin block sections with tumor-infiltrating lymphocytes (TILs) were selected for immunostaining. For those patients with a pathologic complete response on the surgical specimen after NAC, the chemokine receptors were studied on both the core biopsy materials and on surgical specimens (n = 9). Immunohistochemical evaluation was performed with an automatic Ventana BenchMark (Ventana Medical Systems, Tucson, AZ, USA) slide staining device. CXCR4, CXCR5, CCR7, and CCR5 expression was assessed on 5-μm formalin-fixed paraffin-embedded slides. Sections were incubated with primary antibodies for CXCR4 (rabbit mAb; clone D4Z7W, Cell Signaling) at 1:100 dilution, for CXCR5 (rabbit mAb; clone D6L3C, Cell Signaling (Danvers, MA, USA)) at 1:200 dilution, for CCR7 (rabbit polyclonal Ab; and Spring Bioscience (Pleasanton, CA, USA)) at 1:100 dilution, for CCR5 (rabbit polyclonal Ab; clone PA5-29011). 

The chemokine receptors, including CCR5 and CCR7 and CXCR4 and CXCR5, were found to be highly expressed on tumor and tumor-infiltrating lymphocytes (TILs). The mean and median staining percentages of CCR5, CCR7, CXCR4, and CXCR5 on tumor and TILs are shown in Table 1. The median staining percentages of CCR5, CCR7, CXCR4, and CXCR5 expression levels on tumor were 70 (1–90), 50 (2–90), 30 (5–70), and 60 (5–80), respectively, whereas the median staining percentages of CXCR4 and CXCR5 on TILs were 40 (5–70) and 45 (20–90), respectively. In the present cohort, which included patients with TNBC, the chemokine expression pattern on the tumor was mostly cytoplasmic with/without nuclear staining for CCR5 (n = 45, 71.4%), CCR7 (n = 35, 55.6%), CXCR4 (n = 23, 36.5%), and CXCR5 (n = 37, 58.7%). Based on our previous studies and other reports [11,12,13,15,16,31] and the median values of staining percentages of the biomarkers in the present study, any cytoplasmic staining with/without nuclear staining ≥50%, ≥30%, ≥30%, and ≥50% of tumor cells were considered as “high expression” of chemokine receptors CCR5 and CCR7 and CXCR4 and CXCR5, respectively (Table 1 and Figure 1a–d). Moderate or strong intensity, along with the staining percentage, was considered for positive expression of CXCR4 in previous studies, including those mentioned above [11,12,13,15,16,31]. Unlike these studies, Kato et al. considered the intensity of the staining when scoring the expression of chemokine receptors on tumors [14]. However, due to the different antibody clones used in the present study, the intensity of staining was disregarded for the definition of high tumor expression, similar to the study by Andre et al. [17].

**Figure 2:**
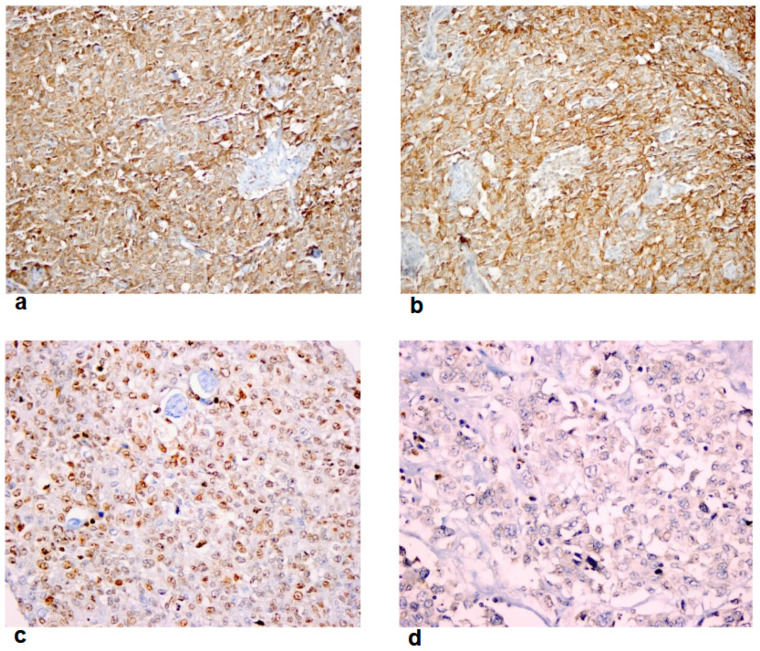
(**a**). High immunohistochemical expression of CCR5 on tumor cells as strong cytoplasmic and nuclear staining (×400, HPF). (**b**). High immunohistochemical expression of CCR7 on tumor cells as strong cytoplasmic staining (×400, HPF). (**c**). High immunohistochemical expression of CXCR4 on tumor cells as moderate cytoplasmic staining (×400, HPF). (**d**). High immunohistochemical expression of CXCR5 on tumor cells (moderate cytoplasmic and nuclear staining) and on tumor-infiltrating lymphocytes (strong nuclear staining) (×400, HPF).

CCR5 and CCR7 were found to be highly expressed in 53.97% and 52.38% of cases, respectively, whereas CXCR4 and CXCR5 were highly expressed in 20.63% and 36.51% of patients, respectively (Figure 2a–d). The majority of the staining pattern of chemokine receptors on tumor were either cytoplasmic or cytoplasmic and nuclear as follows: CCR5 (n = 46, 97.9%), CCR7 (n = 42, 100%), CXCR4 (n = 20, 74.1%), and CXCR5 (n = 37, 78.7%). The high chemokine receptor expressions on tumors, including CCR5, CCR7, and CXCR5, have shown either a moderate or strong staining in the majority of cases as follows: CCR5 (n = 33, 97.1%), CCR7 (n = 32, 97%), and CXCR5 (n = 21, 91.3%). However, of those (n = 13) with a high (≥30%) cytoplasmic or cytoplasmic and nuclear staining expression of CXCR4 on tumors, 9 cases had a weak staining intensity that might be due to the clone used in the present study. Therefore, we did not consider the staining intensity in the present study in the definition of high expression of chemokine receptors on tumors due to the different clones of antibodies used.

A cut-off of ≥1% staining percentage, regardless of staining intensity and staining pattern, was considered positive for CXCR4 and CXCR5 expression on TILs. Of 63 cases, TIL-associated positive staining values for CXCR4 and CXCR5 were 69.84% (n = 44) and 52.38% (n = 33), respectively. The high expression values of chemokine receptors on tumors and any expression and high expression of chemokine receptors on TILs (≥20%) were tested for any significant associations with response to chemotherapy and survival.

## 3. Statistical Analysis

The statistical analyses and figures of the study were performed using the statistical software program SPSS 26 (Statistical Package for Social Sciences; IBM Corp., Armonk, NY, USA) and GraphPad Prism Version 8 Software Program (GraphPad Software San Diego, CA, USA). A *p*-value ≤ 0.05 was considered statistically significant. Categorical variables were evaluated using Fisher’s exact test. Correlations between the expression rates of chemokine receptors on tumors and TILs and RCB-Index scores to assess the response to chemotherapy were determined using Spearman’s test. Disease-free survival (DFS) rates were analyzed while considering local and systemic metastases, and disease-specific survival (DSS) rates were analyzed while considering breast cancer-related mortality. Kaplan–Meier analyses were used for the survival curves test, also known as Mantel–Cox test log-rank test, and this log-rank test was used to compare factors affecting outcomes.

## 4. Results

The mean age was 50.05 ± 13.05 (confidence interval [CI] 95% 46.8–53.3). Demographic and clinicopathological findings are shown in Table 2. Most patients presented with clinically(c) T2-4 disease (cT2; n = 27, 42.9% and cT3; n = 10, 15.9% and cT4; n = 23, 36.5%), whereas few patients had cT1 tumor (n = 3, 4.8%). Similarly, almost all patients presented with axillary positive disease at the initial diagnosis (cN1: n = 37, 58.7%; cN2: n = 16, 25.4%; cN3: n = 7, 11.1%), whereas few had axillary nodal negative disease (cN0: n = 3, 4.8%).

All patients had a diagnosis of locally advanced breast cancer (LABC) and were treated with a multidisciplinary approach, including pre-operative NAC and surgery followed by radiotherapy. The chemotherapy regimen included anthracyclines followed by paclitaxel-containing regimens for all patients. A few patients (n = 3) with poor responses to NAC also received carboplatin. Following NAC, most patients underwent mastectomy (n = 46, 73%) with axillary lymph node dissection (n = 55, 87.3%) with/without sentinel lymph node biopsy (SLNB) due to residual disease in the axilla.

In the definitive pathology, 23 patients (36.5%) were found to have pathologically nodal negativity (ypN0), whereas the remaining 40 were found to have residual axillary nodal disease (ypN1: n = 19, 30.2%; ypN2: n = 12, 19%; ypN3: n = 9, 14.3%). Of those 63 patients, 9 (14.3%) had a pathological complete response (pCR) and Class 0 according to the MDACC Residual Cancer Burden Index. Of those, 54 (85.7%) showed partial response to NAC having MDACC Residual Cancer Burden Index Class I in 4 patients (6.4%), Class II in 21 patients (33.3%), and Class III in 29 patients (46%). Of those with residual cancer in the breast (n = 54), the most common tumor type in surgical specimens was invasive ductal carcinoma in 43 cases (79.6%), invasive lobular carcinoma in 3 patients (5.6%), metaplastic carcinoma in 6 patients (11.1%), and 2 had other breast malignant pathologies (3.7%).

The mean MDACC Cancer Burden Index (RCB) score was 2.71 ± 1.57. Based on the Spearman correlation test, the expression of chemokine receptors CCR5 and CXCR5 on tumor significantly correlated with the RCB scores, indicating a poor response to chemotherapy, whereas the expression of CXCR5 in TILs correlated with a good response to NAC. Patients with high CCR5, CCR7, CXCR4, and CXCR5 expression levels on tumors and high CXCR4 expression levels (≥20%) on TILs were less likely to have a Class 0-I RCB-Index compared to those with a partial pathologic response or Class II-III RCB-Index. Similarly, patients with a pCR were less likely to express CCR5, CCR7, CXCR4, and CXCR5 on tumors, and CXCR4 high expression (≥20%) on TILs in core biopsy tumor materials. However, the difference in expression of CXCR4 on tumors did not reach statistical significance (Table 3).

## 5. Outcomes

**Figure 3:**
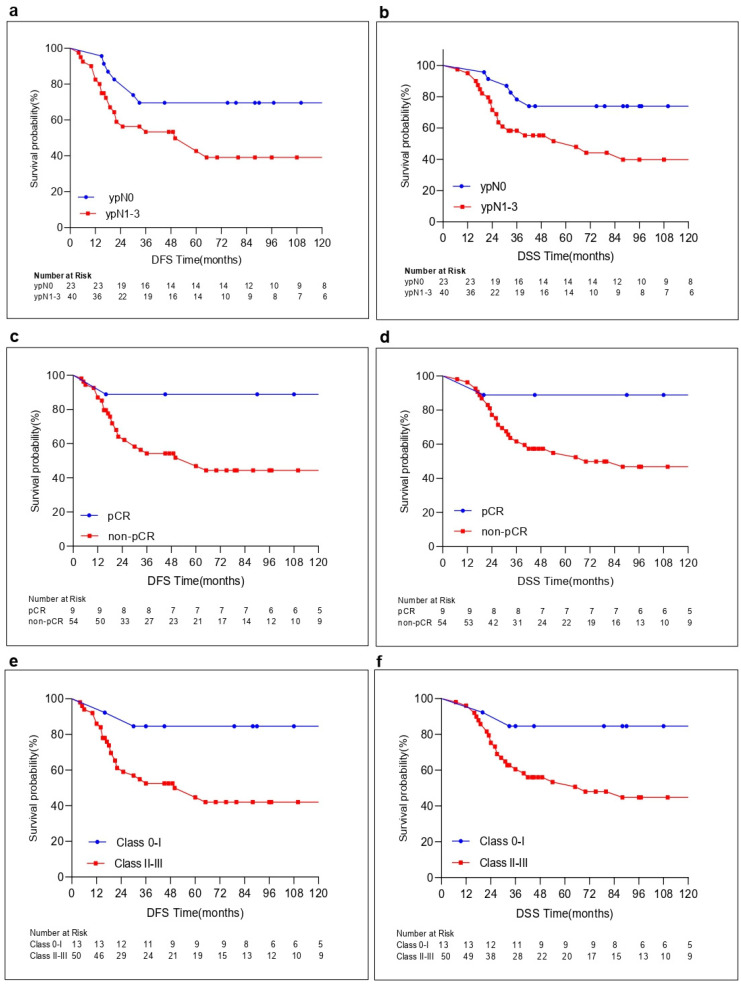
(**a**). 10-year disease-free survival (DFS) in patients with ypN0, 69.6% vs. ypN1-3, 39.1%; *p* = 0.035. (**b**). 10-year disease-specific survival (DSS) in patients with ypN0, 73.9% vs. ypN1-3, 39.8%; *p* = 0.019. (**c**). 10-year DFS in patients with pCR, 88.9% vs. non-pCR, 44.4%; *p* = 0.036. (**d**). 10-year DSS in patients with pCR, 88.9% vs. non-pCR, 46.8%; *p* = 0.049. (**e**). 10-year DFS in patients with MDACC Residual Cancer Burden Index Class 0–I, 84.6% vs. Class II–III, 42.1%; *p* = 0.017. (**f**). 10-year DSS in patients with MDACC Residual Cancer Burden Index Class 0–I, 84.6% vs. Class II–III, 44.8%; *p* = 0.029.

**Figure 4:**
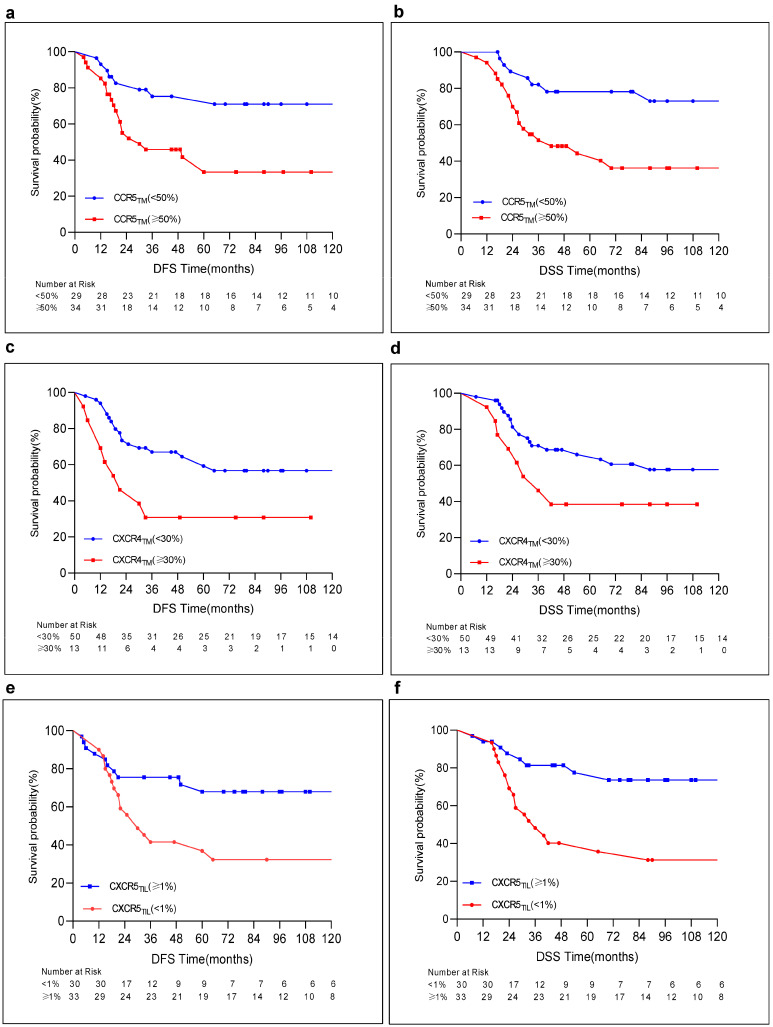
(**a**). 10-year disease-free survival (DFS) in patients with low CCR5 expression on tumor (=CCR5_TM_) (<50%), 71.1%, vs. high CCR5_TM_ (≥50%), 33.4%; *p* = 0.005. (**b**). 10-year disease-specific survival (DSS) in patients with low CCR5_TM_ (<50%), 73.0%, vs. high CCR5_TM_ (≥50%), 36.2%; *p* = 0.004. (**c**). 10-year DFS in patients with low CXCR4 expression on tumor (=CXCR4_TM_) (<30%), 56.7%, vs. high CXCR4_TM_ (≥30%), 30.8%; *p* = 0.017. (**d**). 10-year DSS in patients with low CXCR4_TM_ (<30%), 57.7%, vs. high CXCR4_TM_ (≥30%), 38.5%; *p* = 0.093. (**e**). 10-year DFS in patients with low CXCR5 expression on tumor infiltrating lymphocytes (=CXCR5_TIL_) (<1%), 32.3%, vs. high CXCR5_TIL_ (≥1%), 68%; *p* = 0.015. (**f**). 10-year DSS in patients with low CXCR5_TIL_ (<1%), 31.3%, vs. high CXCR5_TIL_ (≥1%), 73.7%; *p* = 0.001.

Overall, the median follow-up time was 47 months (range: 7–249, interquartile range [IQR]: 18–103). Of 63 patients, 27 (42.9%) died due to distant metastatic disease with a median follow-up of 26 months (IQR, 17–30). The median follow-up time of the survivors was 96 months (IQR, 45–159). Overall, the 5-year and 10-year DFS were 53.3% and 51.2%, and the 5-year and 10-year DSS were 60% and 53.5% in the present cohort. Patients with ypN0, pCR, Class 0–1, and high CXCR5 expression on TILs were more likely to have a better 5-year and 10-year DFS and DSS compared to others (Table 4, Figure 3 and Figure 4). In contrast, patients with high CCR5 and CXCR4 expression levels on tumors were found to have a decrease in 5-year and 10-year DFS and DSS rates compared to those with low expression of CCR5 and CXCR4 on tumors. However, this decrease did not reach statistical significance in 10-year DSS in regards to high CXCR4 expression levels on tumors (Table 4, Figure 4). Notably, no statistically significant difference could be found in 5-year and 10-year DFS and DSS between those with cT1-2 and cT3-4, cN0-1 and cN2-3, high CCR7 or CXCR5 and low CCR7 or CXCR5 expression levels on tumors and, finally, with CXCR4 expression on TILs versus without CXCR4 expression on TILs (Table 4). The statistically significant factors were further evaluated using a Cox regression analysis. At an 8-year median follow-up of survivors, the multivariate analysis in DFS and DSS revealed that patients with ypN-positive, high CCR5_TM_, and CXCR4_TM_ expression levels had an increase in the hazard ratio (HR) compared to others (DFS: HR = 2.655 [1.029–6.852]; DSS: HR = 2.763 [1.008–7.574], DFS: HR = 2.036 (0.805–5.148); DSS: HR = 2.689 (1.020–7.090)), and (DFS: HR = 2.908 (1.080–7.829); DSS: HR = 2.132 (0.778–5.846)), respectively, as shown in Table 5. In contrast, patients without CXCR5_TIL_ expression were found to have an increase in HR compared to those with CXCR5_TIL_ in regard to DFS and DSS (DFS: 2.838 [1.266–6.362]; DSS: 4.211 [1.770–10.016]). These findings indicate a better outcome in these patients with CXCR5 on TILs compared to those without any expression of CXCR5 on TILs in agreement with findings of a reverse correlation between CXCR5 expression on TILs and RCB-Index scores.

## 6. Discussion

Compelling previous evidence has shown that various chemokine receptors were expressed in different types of cancer, including breast cancer, which drives lymphatic and hematogenous spread to critical sites of systemic metastases, including bone, lung, and liver in which their corresponding chemokines were found to be highly expressed [8]. This critical interaction of chemokine receptors and their chemokines triggers both intracellular and extracellular mechanisms that result in the migration, survival, and growth of cancer cells with an increase in angiogenesis, and tumor escape mechanisms. Most studies investigating the key regulatory role of chemokine receptors in tumor progression and spread focus on the CXCR4-CXCL12/CCR7 axis [29]. In the present study, we investigated the expression patterns of CXCR4 and CCR7 along with CCR5 and CXCR5 in locally advanced TNBC patients who were receiving NAC. Here, we demonstrated high expression levels of chemokine receptors on tumors and TILs in patients with TNBC and locally advanced disease. Briefly, chemokine expression on tumor cells was associated with poor outcomes, whereas CXCR5 expression on TILs was found to be a good prognostic factor in the present cohort.

Müller et al. previously reported that the chemokine receptors CXCR4 and CCR7 are found to be highly expressed in human breast cancer cells [8]. The authors demonstrated that the ligand, CXCL12, stimulated the CXCR4-expressing TNBC cell line, MDAMB-231, and led to an increase in the chemotaxis and chemoinvasion of tumor cells toward its ligand. In their experimental studies, inhibition of the CXCL12/CXCR4 interaction with a neutralizing antibody inhibited lymph node and lung metastases [8]. Similarly, a previous study from MD Anderson Cancer Center (MDACC) by Cabioglu et al. reported a high CXCR4 expression of 41% in distant metastatic sites, including bone, lung, liver, and brain of breast cancer patients [12].

In the present study, we found that almost 21% of patients had high CXCR4 expression levels on tumors, and 70% of them also had CXCR4 expression on TILs in patients with TNBC who underwent NAC due to the LABC. The CCR7 chemokine receptor was found to be highly expressed in 52.4% of cases in the present cohort with TNBC. At the MDACC, Cabioglu et al. previously demonstrated that patients with inflammatory breast cancer (n = 44) had 41% (n = 18) high CXCR4 and 23% (n = 10) high CCR7 expression levels based on immunohistochemical staining associated with worse prognosis that was not found to be statistically significant (5-year OS:24.8% for CXCR4 (+) versus 42.3% for CXCR4 (−); *p* = 0.53 and 5-year OS:20% for CCR7 (+) versus 41.9% for CCR7 (−); *p* = 0.24, respectively) [16]. Therefore, despite similar expression levels of CXCR4 in TNBC with LABC in the present cohort and in the cohort of IBC patients at the MDACC, CCR7 expression was more frequently found in the present cohort with TNBC compared to those with IBC.

In early breast cancer, CXCR4 was expressed in 12% of 794 primary tumors. However, CXCR4 expression was found to be associated with increased risk for bone metastases but not with survival [17]. Moreover, patients with node-positive T1 tumors were more likely to express high cytoplasmic CCR7 and CXCR4 staining (CCR7: 21.5% versus 8.5%; *p* = 0.013, and CXCR4: 11.2% versus 5.1%; *p* = 0.113) with different statistical significance compared to those with node-negative T1 tumors [13]. In contrast, nuclear CXCR4 staining was more likely to be detected in lymph node-negative tumors (54.5% versus 37.8%; *p* = 0.018) [13]. Based on these findings, we always considered any cytoplasmic staining as a high expression level of chemokine receptors in all of our analyses. Similarly, Liu et al. compared the expression levels of CXCR4, CCR7, CXCL12, CCL21, and epidermal growth factor receptor (EGFR) in 200 primary breast cancer samples with the corresponding lymph node metastases in a tissue microarray based on immunohistochemistry. They found that the CXCL12 and CCL21 ligands and their corresponding receptors, CXCR4 and CCR7, were highly and significantly expressed in tumor cells with lymph node metastases associated with shorter survival in this cohort [32].

Despite controversial findings regarding the prognostic significance of CXCR4 expression in breast cancer, Yu et al. reported that CXCR4 high expression was associated with a worse prognosis in patients with TNBC [33]. In the present study, we similarly found that patients with ypN-positive (DFS: HR = 2.655 [1.029–6.852]; DSS: HR = 2.763 [1.008–7.574]), high CXCR4_TM_ (DFS: HR = 2.036 [0.805–5.148]; DSS: HR = 2.689 [1.020–7.090]), and high CCR5_TM_ expression (DFS: HR = 2.908 [1.080–7.829]; DSS: HR = 2.132 [0.778–5.846]) had an increased HR compared to others as indicators of poor survival in this patient population. In agreement with these findings, Xu et al. reported in a meta-analysis that included 15 published studies (n = 3104) that CXCR4 expression was found to be a poor indicator of outcome with the HR of 1.65 (95% CI: 1.34–2.03; *p* < 0.00001) for OS and 1.94 (95% CI: 1.42–2.65; *p* < 0.00001) for DFS, respectively [34]. Its expression in the cell, especially in the cytoplasm, was found to be a poor indicator of survival, similar to our findings with the cytoplasmic staining in the IHC in our previous cohort studies. In concordance with the present study, in the meta-analysis by Zhang et al. of 13 studies that included 3865 cases, patients with CXCR4 expression were more likely to be found with lymph node involvement (pooled RR = 1.20, 95% CI: 1.01–1.43), and distant metastasis (pooled relative risk [RR] =1.52; 95% CI: 1.17–1.98) that was associated with poor DFS (RR = 0.77, 95% CI = 0.70–0.86; *p* = 0.554) and overall survival (OS) (RR = 0.70, 95% CI = 0.59–0.83, *p* = 0.329) [35]. Furthermore, Guembarovski et al. analyzed genetic polymorphisms in CXCL12 (rs1801157, G > A) and CXCR4 (rs2228014, C > T) and CXCR4 expression in patients with TNBC by immunohistochemistry [36]. Genetic polymorphisms were analyzed in 59 patients and 150 control women and were found to be correlated negatively with proliferation index Ki67 (*p* = 0.006). CXCR4 immunostaining was evaluated in 37 TNBC patients. CXCR4 expression as membranous staining of tumor cells was correlated positively with histopathological grade (*p* = 0.036) and negatively with lymph node metastasis (*p* = 0.036). Moreover, Shim et al. investigated CXCR4 and CXCL12 expression in 283 patients with TNBC by immunohistochemistry, who presented with operable early-stage breast cancer and were treated with surgery and adjuvant chemotherapy [37]. High cytoplasmic CXCR4 expression was associated with younger age (*p* = 0.008), higher histologic grade (*p* = 0.007), and a better pathologic stage (*p* = 0.045), whereas high CXCL12 expression was associated with bigger tumor size (*p* = 0.045), lymph node metastasis (*p* = 0.005), and advanced pathologic stage (*p* = 0.017). In contrast to the present study and the previous studies, they also reported a better prognosis with less recurrence and improved DFS in patients with cytoplasmic staining. These discrepancies might be due to the different patient populations studied since all patients presented with locally advanced TNBC in the current study, and were treated with NAC followed by surgery. Finally, expression of genes associated with B lymphocyte recruitment and lymphoid infiltration, including CXCL13, CXCR4, and DC-LAMP, was found to be elevated in TNBC compared with other subtypes and normal breast [38]. Similarly, expression of CXCR4 on TILs was detected in the majority of the TNBC samples (70%) in the present cohort.

Recent studies have suggested an interaction between CXCL12/CXCR4 signaling and the CXCL19/CCR7 axis [39]. Hayasaka et al. indicated that CXCL12/CXCR4 signaling promoted breast cancer cell migration and invasion toward CCR7 ligand-expressing intratumoral lymphatic vessels associated with lymph node metastasis [39]. Furthermore, transfection with knockdown CCR7 induced a significant reduction in cell activity and migration capacity in MDA-MB-231 cells [40]. Based on the lncRNA, miRNA, and mRNA expression data of CCR7/CCL19 downloaded from the TCGA database, the 1008 BC samples containing survival data were analyzed. Interestingly, patients with high expression of CCR7 and CCL19 were found to have improved survival compared to those with low expression [40]. There are few reports on the significance of CCR7 expression on human breast cancer cells by immunohistochemistry [41,42,43]. In the study of Vahedi et al., CCR7 expression was detected in 63 (91.4%) of 70 patients with breast cancer. CCR7 expression was significantly associated with disease stage, grade, lymph node metastasis, and perineural and vascular invasion [42]. Similar to the findings with cytoplasmic CXCR4, cytoplasmic CCR7 expression was associated with recurrence in patients with TNBC (n = 133) using tissue microarray by immunohistochemistry [43]. However, no difference could be found in survival between patients with CCR7-low and CCR7-high expression, which is similar to our findings in the present cohort [43].

The current study has shown that CCR5 and CXCR5 were highly expressed on tumors in 54% and 36.5% of cases, and CXCR5 was found to be expressed on TILs in 52.4% of patients diagnosed with locally advanced TNBC. Furthermore, patients with high CCR5, CCR7, CXCR4, and CXCR5 expression levels on tumors and high CXCR4 expressions on TILs were less likely to have a pCR or Class 0-I RCB-Index compared to those with a partial pathological response or Class II-III RCB-Index. Notably, CCR5 expression on tumors was found to be a significant independent prognostic factor among other chemokine receptors associated with poor outcomes. Few reports about the predictive or prognostic role of CCR5 and CXCR5 expressed either on tumors or TILs have been published so far. Previous reports have shown that CCR5 was highly expressed in breast cancer specimens and lymph node metastases, and its expression was found to be correlated with later stages as detected by RT-PCR and immunohistochemistry [44]. Furthermore, the expression of CCR5 in the tumor microenvironment was also demonstrated as a poor prognostic factor in patients with TNBC (n = 184) by immunohistochemistry and from the GEO database [23]. In experimental studies, prominent enhancement has been observed in tumor formation in nude mice injected with CCR5^+^ SUM-159 breast cancer cells compared to those injected with CCR5^−^ breast cancer cells [45]. Finally, Raghavakaimal et al. reported that patients with CCR5^+^ circulating tumor-associated stromal cells were found to have a poor prognosis among patients with metastatic breast cancer [46].

To our knowledge, we report in this study for the first time that CXCR5 expression on TILs was found to be correlated with better chemotherapy response and a good outcome in triple-negative breast cancer. Previous studies demonstrated that the B-cell chemoattractant CXCL13 was recently found to be associated with the CXCR5^+^ T follicular helper cell (T_FH_ cell) infiltration and improved survival in human cancer [47]. T_FH_ cells are described as a subpopulation of CD4^+^ T cells that play a critical role in the development of Ab-producing and memory B cells that might be important in immune response to viral infections [48]. T_FH_ cells were first studied in breast cancer and colon cancer [49,50]. Interestingly, these T_FH_ cells were found as residents of peritumoral lymphoid structures (TLS) in breast cancer and characterized by CXCL13 expression [49]. Furthermore, the CXCL13 single gene was strongly associated with a higher rate of pCR, particularly in the HER2+ subtype in a cohort diagnosed with breast cancer and treated with neoadjuvant chemotherapy (n = 996) supporting the hypothesis that the presence of CXCL13-producing Tfh cells may induce antitumor immune responses associated with improved outcome.

The prognostic significance of CXCL13 in Tfh has been investigated in breast cancer tissue samples by Razis et al. [51]. Of 321 formalin-fixed paraffin-embedded primary tumor tissue samples of patients with early breast cancer treated with chemotherapy, mRNA expressions of CXCL12, CXCL13, and CXCR5 were studied. Interestingly, CXCL13 or CXCR5 expressions showed negative associations with estrogen receptor or microtubule-associated protein tau mRNA expression or dense lymphocytic infiltration, whereas positive associations were found with nuclear grade, and only CXCL13 was positively associated with HER2. In multivariate analysis, they demonstrated increased DFS and decreased hazard ratio with high CXCL13 mRNA expression and increased overall survival and decreased hazard ratio with high CXCL12 expression. Interestingly, in the HER2(+) subgroup, high CXCL13 mRNA expression was significantly found to be associated with improved DFS, whereas high CXCR5 was significantly associated with increased DFS and OS in concordance with our findings showing improved outcomes in patients with CXCR5 expressing TILs.

Noël et al. recently reported that CD4+ Tfh TIL, CD8+ TIL, and TIL-B, colocalizing in TLS, all express the CXCL13 receptor CXCR5. CXCR5^+^ TIL, the target of CXCL13 recruitment, was analyzed in a cohort of invasive ductal BC patients (n = 168) and was more frequently observed in HER2-positive and TNBC. The CXCL13 receptor CXCR5 expressing TILs as described activated, functional Th1-oriented Tfh TILs (PD-1hiICOSint phenotype) was reported to play a role in immunoglobulin and interferon-gamma (IFN-γ) production that was associated with a good clinical outcome [52]. Of note, a cytotoxic T cell subpopulation expressing high levels of PD-1 and CXCR5 was recently described in the lymph nodes of breast cancer patients with unknown significance [53]. These particular cells might also be a target of PD-1/PD-L1 blockade-based immunotherapeutic approaches.

In colorectal cancer (CRC), CXCL13 was detected in both T cells and tumor cells, and *CXCL13* deletion characterized with fewer infiltrating T_FH_ and B cells was associated with a higher recurrence rate and poorer survival [50]. High expression of B cell markers, including CD19 and Tfh cell markers, CXCL13 and CXCR5, were significantly correlated with an increased DFS. Further, the prognostic significance was also tested in murine orthotopic CRC models. Mouse CRC cell line MC38 was injected endoscopically into the colonic submucosa of syngenic C57Bl/6 mice that are wild-type and Rag1^−/−^. Tumors grown in wild-type mice expressed higher levels of markers of the adaptive immune response, including CD8, CD4, CXCR5, and CXCL13, compared to those Rag1^−/−^ mice. As expected, the growth of orthotopic MC38 tumors was significantly accelerated in Rag1^−/−^ mice in comparison to wild-type mice. The authors further validated these findings in other mouse models using CXCR5^−/−^ mice that were subjected to endoscopic orthotopic injection of tumor cells. Similarly, these mice showed accelerated tumor growth compared to the wild-type mice. In contrast, endoscopic recombinant CXCL13 injection within the colonic submucosa of wild-type mice inhibited tumor growth in wild-type mice. Therefore, all these findings suggest CXCR5 and CXCL13 as good prognostic markers of TILs against cancer cells as both confirmed in animal and human studies associated with a better clinical outcome.

However, the prognostic significance of CXCR5 and CXCL13 expressions on tumors might have different prognostic significance, unlike their expression on TILs. Jiang et al. investigated the expression of CXCL13 and CXCR5 expression in 133 patients with breast cancer by quantitative real-time polymerase chain reaction (qRT-PCR) and immunohistochemical staining [18]. The expression of CXCL13 and CXCR5 was significantly higher in breast cancer tissue than in normal breast tissues. High expression of CXCL13 and CXCR5 in breast cancer tissues was found to be associated with increasing stage, lymph node metastasis, distant metastasis, and disease stage, but not with HER2 status, histological type, or tumor size. Finally, the 5-year survival was negatively correlated with distant metastasis and expression of CXCL13 and CXCR5. We similarly found a decreased DFS and DSS in patients with high CXCR5 expression; however, these findings were not found to be statistically significant. Finally, the expression of novel markers, including CXCR5, has been recently investigated in cancer stem-like cells (CSCs) in 125 patients with breast cancer [54]. Gene expression analysis from in vitro mammosphere formation revealed IFNG, CXCR5, CD40LG, TBX21, and IL2RG to be associated with the CSC phenotype and also displayed prognostic value for patients with breast cancer. Further analyses showed that the expression of IFNG and CXCR5 were significantly associated with tumor size (*p* = 0.032 and *p* = 0.047, respectively), and their expression was lower in invading tumors (T4). In the TCGA BRCA dataset, similarly, the authors have demonstrated significant associations between lower expression of IFNG, CD40LG, and CXCR5 and larger tumor sizes or adjacent tissue invasion (*p* = 0.01, *p* = 0.03, and *p* = 0.01, respectively). Therefore, more studies are needed to explore the prognostic significance of CXCR5 expression on tumors and TILs.

Furthermore, gene set enrichment analyses indicated that patients with residual TNBC following neoadjuvant durvalumab and chemotherapy in the GeparNuevo randomized trial were more likely found to express CCL-3, -4, -5, -8, -23, CXCL-1, -3, -6, -10, and interleukin (IL)1, -23, -27, -34. In concordance with our findings, patients with pCR were interestingly more likely found to express IFNγ, IL2, -12, -21, chemokines CXCL-9, -13, CXCR5, and activated T and B-cell markers [55]. Furthermore, Blaye et al. identified eight genes on TILs in tumor-associated microenvironment in patients with TNBC and residual disease after NAC that were associated with metastatic relapse (BLK, GZMM, CXCR6, LILRA1, SPIB, CCL4, CXCR4, SLAMF7) in agreement with our findings showing CXCR4_TIL_ expression was related with poor response to NAC [56]. In contrast, patients with pCR had significantly more effector antitumoral immune cells. Lack of immune activation after NAC may be associated with a high risk of distant relapse. Therefore, immunotherapies targeting receptors associated with a poor prognostic immune signature on TILs could lead to an improvement in TNBC prognosis.

Targeting the CXCL12-CXCR4/CXCR7 signaling axis has been a promising approach for tumor therapy in recent years [29,57]. The human immunodeficiency virus (HIV)-related chemokine receptor (HIVrCR) antagonists were proposed for the treatment of hematological and solid malignancies, including leukemia, lymphoma, breast cancer, pancreatic cancer, and colorectal cancer. These medicines targeting CXCR4 can be categorized as non-peptide CXCR4 antagonists such as AMD3100, peptide CXCR4 antagonists such as BTK140, and CXCR4 antibodies such as ulocuplumab, respectively. Chemokine receptor antagonist (CRA) drugs such as plerixafor (CXCR4 antagonist) or maraviroc (CCR5 antagonist) have been shown to induce suppression of cancer cell proliferation, migration, and metastasis [58,59]. AMD3451 is the first reported dual CXCR4/CCR5 antagonist [60], whereas other antagonists, including NF279, penicillixanthone A, and GUT-70, have also shown a dual effect [61]. Thus, the CRA-mediated blockade of these axes could be used as an alternative or complementary therapy to improve the outcome of patients with tumors resistant to chemotherapy [62]. There are few antagonists targeting CXCR7 in preclinical models, such as small molecules CCX266, CCX662, CCX733, and CCX771 [29]. Currently, AMD3100 has been the only drug approved for clinical treatment. However, combining CXCR4 or CCR5 antagonists with anti-PD1 immunotherapy has shown promising results in clinical studies in patients with colorectal cancer and pancreatic ductal adenocarcinoma [63,64]. Future studies are needed to explore the efficacy of chemokine receptor antagonists and their interaction with immunotherapy.

## 7. Conclusions

These results demonstrate that chemokine receptors were highly expressed in patients with locally advanced TNBC who had partial responses to NAC. Expression of CXCR4 and CCR5 on tumor was found to be associated with poor prognosis in this cohort with TNBC, and CXCR5 on tumor was associated with chemotherapy resistance. Further studies should be performed targeting the chemokine receptors CXCR4 and CCR5 and CXCR5 on tumors with a poor prognostic signature in addition to chemotherapy regimens that might improve the outcome in patients with locally advanced TNBC [29,57].

## Figures and Tables

**Table 1:** Expression profile of chemokine receptors in patients with triple-negative breast cancer with locally advanced breast cancer.

N = 63	Mean Value (%) +/− sd	Median Value (Min–Max)	Any Expression Rate	Positive Expression
CCR5-_TM_	66.60 ± 21.18	70 (1–90)	74.60% (47/63)	≥50%, any cytoplasmic staining
CCR7-_TM_	52.49 ± 24.38	50 (2–90)	66.67% (42/63)	≥30%, any cytoplasmic staining
CXCR4-_TM_	33.60 ± 18.85	30 (5–70)	42.86% (27/63)	≥30%, any cytoplasmic staining
CXCR4-_TIL_	40.60 ± 20.33	40 (5–70)	69.84% (44/63)	≥1%, ≥20% (high expression)
CXCR5-_TM_	48.85 ± 22.37	60 (5–80)	74.60% (47/63)	≥50%, any cytoplasmic staining
CXCR5-_TIL_	45.15 ± 15.24	45 (20–90)	52.38% (33/63)	≥1% = ≥20%

The mean values of biomarker expressions were calculated for those patients with any receptor expression on tumor (TM) or tumor-infiltrating lymphocytes (TILs). Patients without any chemokine receptor expression were excluded from these analyses.

**Table 2:** Clinical and pathological characteristics of patients with triple-negative breast cancer treated with neoadjuvant chemotherapy (NAC).

Clinical Characteristics	n (%) (N = 63)
**Premenopausal**	28 (44.4%)
**Postmenopausal**	35 (55.6%)
**Age**	
≤40	23 (36.5%)
>40	40 (63.5%)
≤50	34 (54%)
>50	29 (46%)
**Clinical TNM-stage**	
cT1	3 (4.8%)
cT2	27 (42.9%)
cT3	10 (15.9%)
cT4	23 (36.5%)
cN0	3 (4.8%)
cN1	37 (58.7%)
cN2	16 (25.4%)
cN3	7 (11.1%)
**Surgery after NAC**	
Breast-conserving surgery + sentinel lymph node biopsy	4 (6.3%)
Breast-conserving surgery +/− sentinel lymph node biopsy + axillary lymph node dissection	13 (19.6%)
Mastectomy + sentinel lymph node biopsy	4 (6.3%)
Mastectomy +/− sentinel lymph node biopsy + axillary lymph node dissection	42 (66.7%)
ypN0	23 (36.5%)
ypN(+)	40 (63.5%)
MDARB-Index	
Class 0	9 (14.3%)
Class I	4 (6.3%)
Class II	21 (33.3%)
Class III	29 (46.0%)

MDARB-Index = MD Anderson Residual Cancer Burden Index.

**Table 3:** Associations of biomarker expressions with neoadjuvant chemotherapy response in TNBC with locally advanced disease along with correlations with MD Anderson Residual Cancer Burden Index (MDARB-Index).

	MDARCB-Index (*R/*p*-Value)	Biomarker Expression	% (N = 63)	Class 0 (pCR) vs. Class I-III	*p*-Value	Class 0–I vs. Class II–III	*p*-Value
**CCR5-_TM_**	0.617/ <0.0001	≥50%	34 (53.97%)	0/9 vs. 34/54	0.0001	0/13 vs. 34/50	0.0001
**CCR7-_TM_**	0.188/ 0.141	≥30%	33 (52.38%)	1/9 vs. 32/54	0.010	2/13 vs. 31/50	0.004
**CXCR4-_TM_**	0.211/ 0.097	≥30%	13 (20.63%)	0/9 vs. 13/54	0.184	0/13 vs. 13/50	0.053
**CXCR4-_TIL (≥1%)_**	0.011/0.933	≥1%	44 (69.84%)	6/9 vs. 38/54	0.999	8/13 vs. 36/50	0.508
**CXCR4-_TILhigh (≥20%)_**	N.A.	≥20%	36 (57.14%)	2/9 vs. 34/54	0.028	4/13 vs. 32/50	0.057
**CXCR5-_TM_**	0.339/ 0.007	≥50	23 (36.51%)	0/9 vs. 23/54	0.020	0/13 vs. 23/50	0.002
**CXCR5-_TIL_**	−0.349/ 0.005	≥1%	33 (52.38%)	7/9 vs. 26/54	0.152	9/13 vs. 24/50	0.221

*R: Spearman Correlations. TM = tumor; TIL = tumor-infiltrating lymphocytes.

**Table 4:** Outcome of patients according to clinicopathological features and chemokine receptor expression patterns.

	5-Year DFS (%)	*p*-Value	10-Year DFS (%)	*p*-Value	5-Year DSS (%)	*p*	10-Year DSS (%)	*p*
**cT (clinical = physical exam&radiological evaluation)**		0.651		0.527		0.893		0.760
cT1-2 (n = 30)	54.7		54.7		58.5		54.3	
cT3-4 (n = 33)	51.7		47.8		61.6		52.7	
**cN**		0.651		0.770		0.133		0.320
cN0-1 (n = 40)	54.3		50.9		65.5		55	
cN2-3 (n = 23)	50.2		50.2		50.1		50.1	
**Axillary response**		0.052		0.035		0.067		0.019
ypN0 (n = 23)	69.6		69.6		73.9		73.9	
ypN1-3 (n = 40)	42.7		39.1		51.5		39.8	
**MDARB-Index**		0.044		0.036		0.089		0.049
Class 0 (pathologic CR) (n = 9)	88.9		88.9		88.9		88.9	
Class I-III (n = 53)	46.9		44.4		54.9		46.8	
		0.022		0.017		0.061		0.029
Class 0-I (n = 13)	84.6		84.6		84.6		84.6	
Class II-III (n = 50)	44.7		42.1		53.4		44.8	
**Biomarker expressions**								
**CCR5-_TM_**		0.003		0.005		0.008		0.004
<50% (n = 28)	75.3		71.1		78.2		73	
≥50% (n = 35)	33.4		33.4		44.3		36.2	
**CCR7-_TM_**		0.084		0.120		0.075		0.104
<30% (n = 30)	64.8		60.8		71.7		63	
≥30% (n = 33)	42.9		42.9		49.7		45.6	
**CXCR4-_TM_**		0.013		0.017		0.048		0.093
<30% (n = 50)	59.3		56.7		66		57.7	
≥30% (n = 13)	30.8		30.8		38.5		38.5	
**CXCR4-_TIL_**		0.744		0.846		0.644		0.961
<1% (n = 19)	52.1		52.1		57.4		57.4	
≥1% (n = 44)	53.6		50.6		61.2		51.7	
**CXCR4-_TIL_**		0.406		0.482		0.636		0.527
<20% (n = 27)	62		57.2		65.5		60.4	
≥20% (n = 36)	46.7		46.7		56.1		49.1	
**CXCR5-_TM_**		0.440		0.513		0.570		0.563
<50% (n = 40)	58.8		55.7		64		57	
≥50% (n = 23)	42.7		42.7		52.7		46.9	
**CXCR5-_TIL_**		0.028		0.015		0.004		0.001
<1% (n = 30)	36.9		32.3		40.2		31.3	
≥1% (n = 33)	68		68		77.5		73.7	

MDARB-Index = MD Anderson Residual Cancer Burden Index, TM = tumor; TIL = tumor-infiltrating lymphocytes.

**Table 5:** Multivariate Cox regression analysis in TNBC with locally advanced disease treated with neoadjuvant chemotherapy.

	Disease Free Survival		Disease Specific Survival	
Factors	Hazard Ratio (95% CI)	*p* Value	HR (95% CI)	*p* Value
**Pathologic Complete Response (axilla)**		0.044		0.048
ypN0	Reference (1)		Reference (1)	
ypN-positive	2.655 (1.029–6.852)		2.763 (1.008–7.574)	
**CCR5** **-_TM_**		0.133		0.046
<50%	Reference (1)		Reference (1)	
≥50%	2.036 (0.805–5.148)		2.689 (1.020–7.090)	
**CXCR4** **-_TM_**		0.035		0.141
CXCR4-_TM_ (−)	Reference (1)		Reference (1)	
CXCR4-_TM_ (+)	2.908 (1.080–7.829)		2.132 (0.778–5.846)	
**CXCR5-_TIL_**		0.011		0.001
CXCR5-_TIL_ (−)	2.838 (1.266–6.362)		4.211 (1.770–10.016)	
CXCR5-_TIL_ (+)	Reference (1)		Reference (1)	

Hazard ratios (HR) are presented with their 95% confidence interval (CI) and the *p*-value. TM = tumor; TIL = tumor-infiltrating lymphocytes.

## Data Availability

The datasets used and/or analyzed during the current study are available from the corresponding author upon reasonable request.
